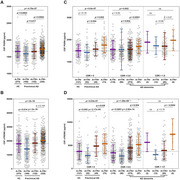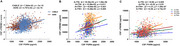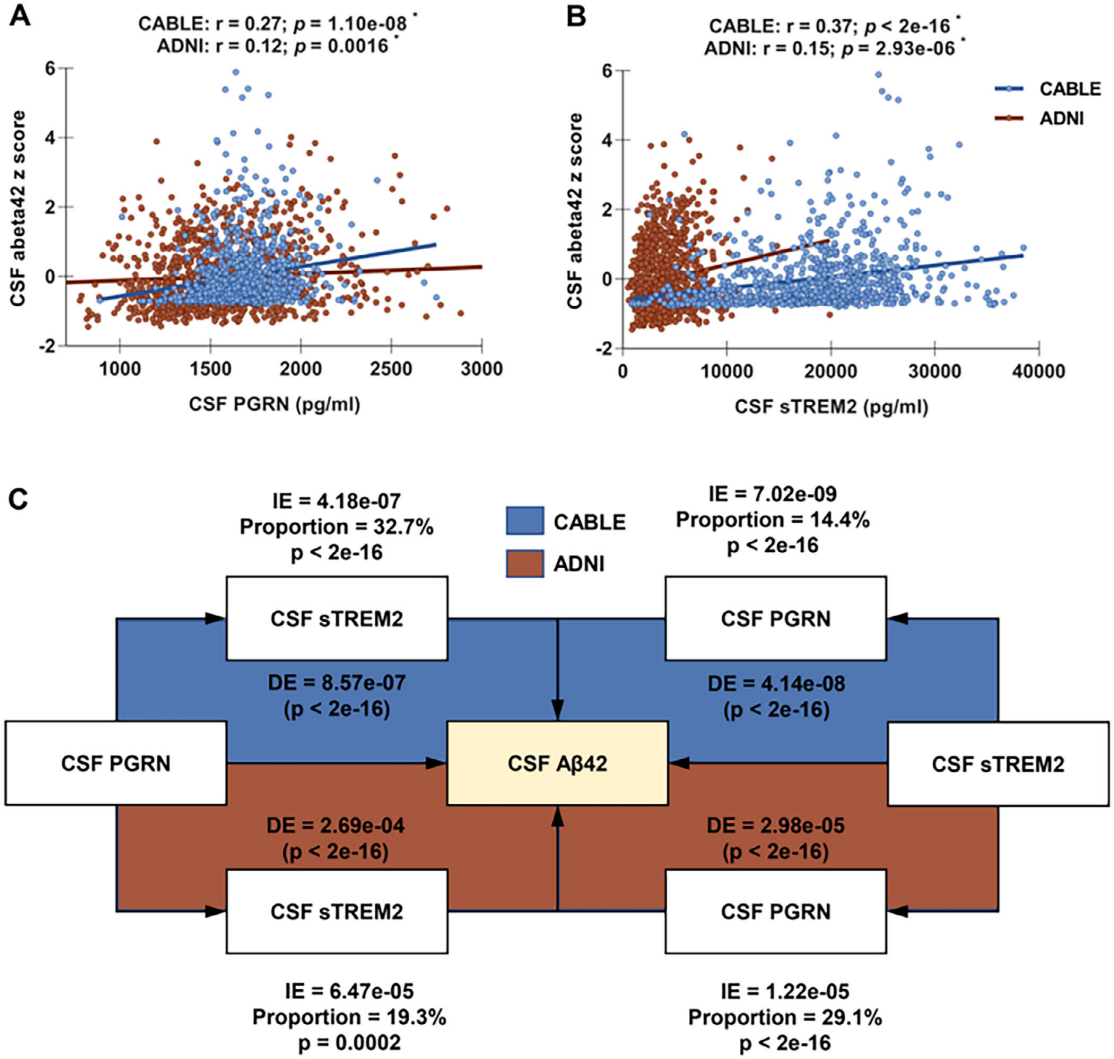# Relationships of PGRN with sTREM2 in AD continuum and non‐AD pathophysiology and their reciprocal roles in modulating amyloid pathology: two population‐based study

**DOI:** 10.1002/alz70856_101874

**Published:** 2025-12-25

**Authors:** Liangyu Huang

**Affiliations:** ^1^ Qingdao Municipal Hospital, Qingdao, Shandong, China

## Abstract

**Background:**

Progranulin (PGRN) and soluble triggering receptor expressed on myeloid cells‐2 (sTREM2) are emerging markers involved with Alzheimer's disease (AD). This study aimed to explore the associations of cerebrospinal fluid (CSF) PGRN and sTREM2 with AD biomarkers across AD spectrum and non‐AD pathophysiology, focusing on their interplay in mediating Aβ pathology.

**Method:**

We analyzed cross‐sectional data of 905 participants (mean age = 62.0) from the Chinese Alzheimer's Biomarker and Lifestyle (CABLE) cohort and 973 (mean age = 73.1) from the Alzheimer's Disease Neuroimaging Initiative (ADNI), classified using the A/T/N biomarker framework. One‐way analyses of covariance, followed by Bonferroni corrected post hoc comparisons, were used to assess whether CSF PGRN and sTREM2 differed across biomarker profiles and clinical stages. Multiple linear regression models were used to assess the relationships of PGRN with sTREM2 as well as their associations with CSF Aβ_1‐42_, *p*‐tau_181_, and T‐tau. Causal mediation analyses were performed to explore the reciprocal relationships between sTREM2 and PGRN in modulating Aβ pathology, with 10,000 bootstrapped iterations.

**Result:**

In both cohorts, CSF levels of both PGRN and sTREM2 were significantly higher in A‐/TN+ group but lower in A+/TN‐ group. CSF PGRN was associated with higher sTREM2 across varied A/T/N profiles (*p* < 2×10^‐16^). Bidirectional mediation roles were revealed between PGRN (mediation percentage 14.4% in CABLE and 29.1% in ADNI) and sTREM2 (32.7% in CABLE and 19.3% in ADNI) in modulating the relationships with Aβ pathology (*p* < 0.0001).

**Conclusion:**

These findings suggested that the interplay between lysosome function and microglia‐related neuroinflammation could play important roles in amyloid metabolism.